# Correlated color temperature is not a suitable proxy for the biological potency of light

**DOI:** 10.1038/s41598-022-21755-7

**Published:** 2022-11-23

**Authors:** Tony Esposito, Kevin Houser

**Affiliations:** 1 Lighting Research Solutions LLC, Philadelphia, PA USA; 2 School of Civil and Construction Engineering, Oregon State University, Corvallis, OR USA; 3 Pacific Northwest National Laboratory, Portland, OR USA

**Keywords:** Lasers, LEDs and light sources, Civil engineering

## Abstract

Using a simulation based on a real, five-channel tunable LED lighting system, we show that Correlated Color Temperature (CCT) is not a reasonable predictor of the biological potency of light, whether characterized with CIE melanopic Equivalent Daylight Illuminance (mel-EDI), Equivalent Melanopic Lux (EML) (a scalar multiple of mel-EDI), or Circadian Stimulus (CS). At a photopic corneal illuminance of 300 lx and *R*_f_ ≥ 70, spectra can vary in CS from 17 to 41% across CCTs from 2500 to 6000 K, and up to 23% at a single CCT, due to the choice of spectrum alone. The CS range is largest, and notably discontinuous, at a CCT of 3500 K, the location of the inflection point of the CS model. At a photopic corneal illuminance of 300 lx and *R*_f_ ≥ 70, mel-EDI can vary from 123 to 354 lx across CCTs from 2500 to 6000 K and can vary by up to 123 lx at a fixed CCT (e.g., 196 to 319 lx at 5000 K). The range of achievable mel-EDI increases as CCT increases and, on average, decreases as color fidelity, characterized with IES TM-30 *R*_f_, increases. These data demonstrate that there is no easy mathematical conversion between CS and mel-EDI when a spectrally diverse spectra set of spectral power distributions is considered.

## Introduction

Light is employed in the built environment to support human *visual* responses, including visual performance, visual comfort, color rendition, control of glare and flicker, psychological reinforcement, and aesthetic integration. These visual goals are balanced while minimizing energy use, which includes considerations of luminous efficacy, lighting power density, and controls.

In recent years, there has been growing understanding of the influence of light and lighting on non-visual biological responses, including circadian phase shifting^1,2^, alertness^3,4^, melatonin suppression^2,5^, pupillary response^6,7^, heart rate^8,9^, and body temperature^8,9^. With this ever-expanding body of knowledge, it has become increasingly important to design and specify lighting that also acknowledges lighting’s potential to influence human non-visual response^10,11^. Lighting industry constituencies that include manufacturers, specifiers, researchers, and standards bodies are working to accommodate this new reality.

Correlated color temperature (CCT), which describes the visual “warmness” or “coolness” of the color appearance of light when viewed directly, is readily available information in product specifications. CCT has sometimes been employed as a shorthand proxy for light’s biological potency. Importantly, doing so is based on misunderstandings of the underlying meaning and appropriate uses of CCT.

A notable example comes from the Council on Science and Public Health (CSAPH) which has warned against using high CCT lighting in the outdoor nighttime environment^12^. This warning led to the adoption of American Medical Association (AMA) policy H-135.927^13^ that “encourages the use of 3000 K or lower lighting for outdoor installations such as roadways”, a recommendation that is currently in effect. In response to the AMA policy, the Lighting Research Center^14^, Illuminating Engineering Society (IES)^15^, and Houser^16^ argued that CCT is just one aspect of lighting quality and that CCT is inadequate for evaluating potential health outcomes of light exposure.

The goal of this study is to provide numerical support for the assertion that CCT is not suitable for predicting the biological potency of light. Using a simulation based on a real 5-channel LED lighting system, we explicitly demonstrate that CCT is not a suitable proxy for prevailing measures of the biological potential of light, by showing that significant and practically meaningful variation in those measures exists at any fixed value of CCT and photopic illuminance.

## Background

### Correlated Color Temperature (CCT)

Correlated Color Temperature (CCT, symbol: *T*_cp_), describes the “temperature of a Planckian radiator having the chromaticity nearest the chromaticity associated with the given spectral distribution…”^17^. The CCT of a light source is computed as the temperature (in kelvin) of the Planckian radiator with the closest chromaticity. The computation is performed in the CIE 1960 *uv* chromaticity diagram, which is officially the (u’, 2/3v’) uniform chromaticity scale (UCS) diagram because the 1960 UCS diagram has been obsoleted by CIE and is not used for any purpose other than computing CCT. CCT is derived entirely from a light source’s spectral power distribution (SPD). CCT applies to light sources that appear nominally white; generally, light sources with high CCT appear “cool” (a “blue” tinted appearance) and light sources with low CCT appear “warm” (an “orange” tinted appearance).

**Figure 1:**
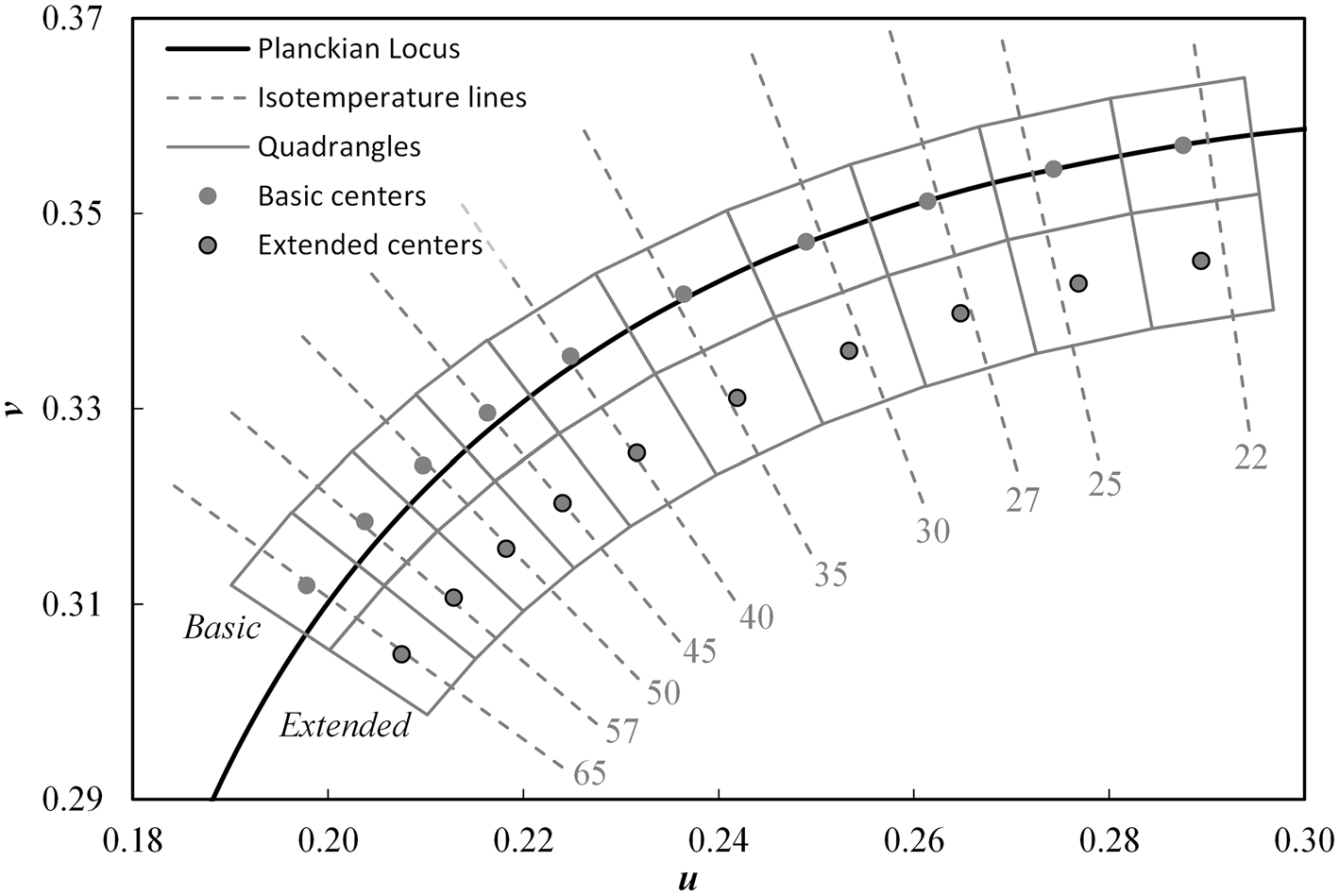
An enlarged portion of the CIE 1960 *uv* diagram showing select isotemperature lines and the ANSI C78.377-2017 *Basic* and *Extended* CCT quadrangles. Labels indicate the CCT with the trailing double zero removed to avoid clutter (e.g., “30” = 3000 K).

SPDs with equal CCT fall along a line perpendicular to the Planckian locus—i.e., isotemperature lines—in the CIE 1960 *uv* chromaticity diagram (Fig. 1). Along an isotemperature line, the distance *D*_uv_ describes how far the chromaticity of a spectral power distribution (SPD) is above (in the nominally “yellow/green” direction) or below (in the nominally “pink” direction) the Planckian locus at the same temperature. *D*_uv_ values above the Planckian locus are positive; values below are negative^18^. CCT and *D*_uv_ form a two-measure system for expressing the chromaticity of nominally white light^19^.

Chromaticity tolerances are provided by ANSI^18^ to “ensure high quality white light” and to “categorize chromaticities…so that white light…can be communicated to consumers.” The ANSI specification takes the form of quadrangles in chromaticity space encapsulating chromaticities that can be considered to have the corresponding nominal CCT. Nominal CCTs in the ANSI framework include 2200 K, 2500 K, 2700 K, 3000 K, 3500 K, 4000 K, 4500 K, 5000 K, 5700 K, and 6500 K. The *Basic* ANSI quadrangles are positioned along the Planckian locus. A set of additional quadrangles—the *Extended* quadrangles—are located below the *Basic* quadrangles and below the Planckian locus; see ANNEX E of ANSI^18^ for more information. Figure 1 shows the *Basic* and *Extended* ANSI quadrangles in the CIE 1960 *uv* chromaticity diagram.

CCT is regularly reported in lighting product specifications making it readily available and frequently specified. Importantly, CCT is a measure of the visual perception of illumination and is itself only one-half of a two-measure system (CCT and *D*_uv_) to specify a light source’s chromaticity. CCT is not a unique descriptor because many different SPDs can share the same CCT, even at the same *D*_uv_.

### Available measures for quantifying biological potency

The emerging metrology in quantifying the biological potency of optical radiation is based on two distinct approaches: (1) the nocturnal suppression of the hormone melatonin^20–24^, and (2) the direct excitation of retinal photopigments^25–27^.

The only international standard for quantifying the biological potency of light, the *CIE System for Metrology of Optical Radiation for ipRGC-Influenced Responses to Light* (CIE S026)^26^, is based on photopigment excitation. CIE S026 uses a system of photometric equivalence that describes the amount of radiation conforming to standard daylight (D65) that is needed to produce equivalent photopigment stimulation—quantified by the respective opsin-based photopigments—as a test light source. Photopigments and their respective photoreceptors are specified by the “α” in “α-opic” whereby melanopsin, the photopigment in the ipRGC photoreceptors, is specified as “melanopic”. Some have argued that biological potency is most relevant to melanopsin^28^, and melanopsin is the primary opsin-based photopigment considered from the CIE S026 framework in this analysis.

The first step to determine melanopic photometric equivalence is to compute the *melanopic* Daylight Efficacy Ratio (mel-DER), which is the ratio of melanopic luminous efficacies of the test light source and D65. Mel-DER is unitless.

1 $$\text{mel-DER}=\frac{\mathrm{Melanopic \,\,Luminous \,\,Efficacy\,\, of\,\,}\mathrm{Test\,\, Light \,\,Source}}{\mathrm{Melanopic\,\, Luminous \,\,Efficacy \,\,of}\,\,\mathrm{D}65}$$

Next, mel-DER is multiplied by a specified photopic corneal illuminance (E) to produce the *melanopic* Equivalent Daylight Illuminance (mel-EDI) which describes the illuminance of D65 that provides equal melanopic activation as the test source. Mel-EDI is an equivalent illuminance and has the unit lux.

2 $$\text{mel-EDI}=\mathrm{E}*(\text{mel-DER})$$

As an example, a blackbody radiator at 3500 K has a mel-DER of 0.62. At a photopic illuminance of 300 lx, this equals a mel-EDI of 186 lx. Interpretation: 186 lx of daylight at 6500 K is required to produce the same melanopic activation as 300 lx of blackbody radiation at 3500 K. Said another way, at illuminance levels that are above threshold and below saturation, blackbody radiation at 3500 K is 62% as effective at stimulating melanopsin as daylight at 6500 K.

Other measures based on excitation of retinal photopigments preceded CIE S026 (see, for example, Miller and Irvin^28^), with perhaps the most popular method published by Lucas et al.^25^ and adopted by the WELL Building Standard^27^. The process begins with computation of the Melanopic Ratio (MR), which is the quotient of a light source’s melanopic and photopic content, multiplied by 1.218 to normalize MR to a value of 1.0 for an Equal Energy (EE) spectrum^27^. An MR of 1.0 indicates that a light source has the same melanopic-to-photopic ratio as an equal energy illuminant; a value greater than 1.0 indicates a higher melanopic-to-photopic ratio than an EE spectrum, and vice versa. MR is unitless.

3 $$\mathrm{MR}=\frac{\mathrm{Melanopic \,\,content}}{\mathrm{Photopic \,\,content}}*1.218$$

MR is a scalar multiple of mel-DER.

4 $$\mathrm{MR}=1.103*(\text{mel-DER})$$

MR can be multiplied by a specified illuminance (E) to determine equivalent melanopic lux (EML). EML has the unit “melanopic lux” or “m-lux”. Note that melanopic lux is not an SI unit and has no standardized interpretation.

5 $$\mathrm{EML}=\mathrm{E}*\mathrm{MR}$$

Because MR and EML are scalar multiples of mel-DER and mel-EDI, respectively, and CIE’s mel-DER and mel-EDI are standardized measures, only mel-DER and mel-EDI will be reported in this study.

*Circadian Stimulus* (CS) is a measure of nocturnal melatonin suppression proposed by Rea et al.^20–23^. Computing CS begins with computing *Circadian Light* (CL_A_), a model of biological potential that is based on a hypothesized retinal circuity that changes its sensitivity based on the “blue”–“yellow” balance in a light source’s spectrum (henceforth referred to as the b-y spectral opponency). CL_A_ “values are normalized so that a stimulus with a spectral power distribution defined by CIE Illuminant A (a blackbody radiator at 2856 K) having a photopic illuminance at the cornea of 1000 lx equals a CL_A_ value of 1000”^20^.

The b-y spectral opponency of the CS model is essential to interpreting the results of this present work. When a light source contains proportionally more short-wavelength radiation, “blue wins” (henceforth referred to as “rBY+”), and CL_A_ is derived by a hypothesized combination of melanopsin and the traditional cone photoreceptors, where the absolute sensitivity of the cone contribution is mediated by the bleaching of the rod photoreceptors. When a light source contains proportionally more long-wavelength radiation, “yellow wins” (henceforth referred to as “rBY-”), and CL_A_ is derived by melanopsin alone^23^. The percentage of melatonin suppression is then determined by fitting CL_A_ values to a four-parameter logistic function. CS is equal to the percentage of nocturnal melatonin suppression and ranges from 0.00 to 0.70 (0–70% nocturnal melatonin suppression). The CS model considers spectrum and intensity and assumes one hour of exposure. CS is the primary measure used in UL DG 24480 *Design Guideline for Promoting Circadian Entrainment with Light for Day-Active People* (UL 24480)^24^, though UL 24480 allows alternate compliance paths.

This study is not intended to validate (or invalidate) Circadian Stimulus^23^, melanopic Equivalent Daylight Illuminance^26^, or Equivalent Melanopic Lux (EML)^27^. Instead, these metrics were used as proxies for the magnitude of the biological potential of a light stimulus.

## Methodology

### Apparatus

**Figure 2:**
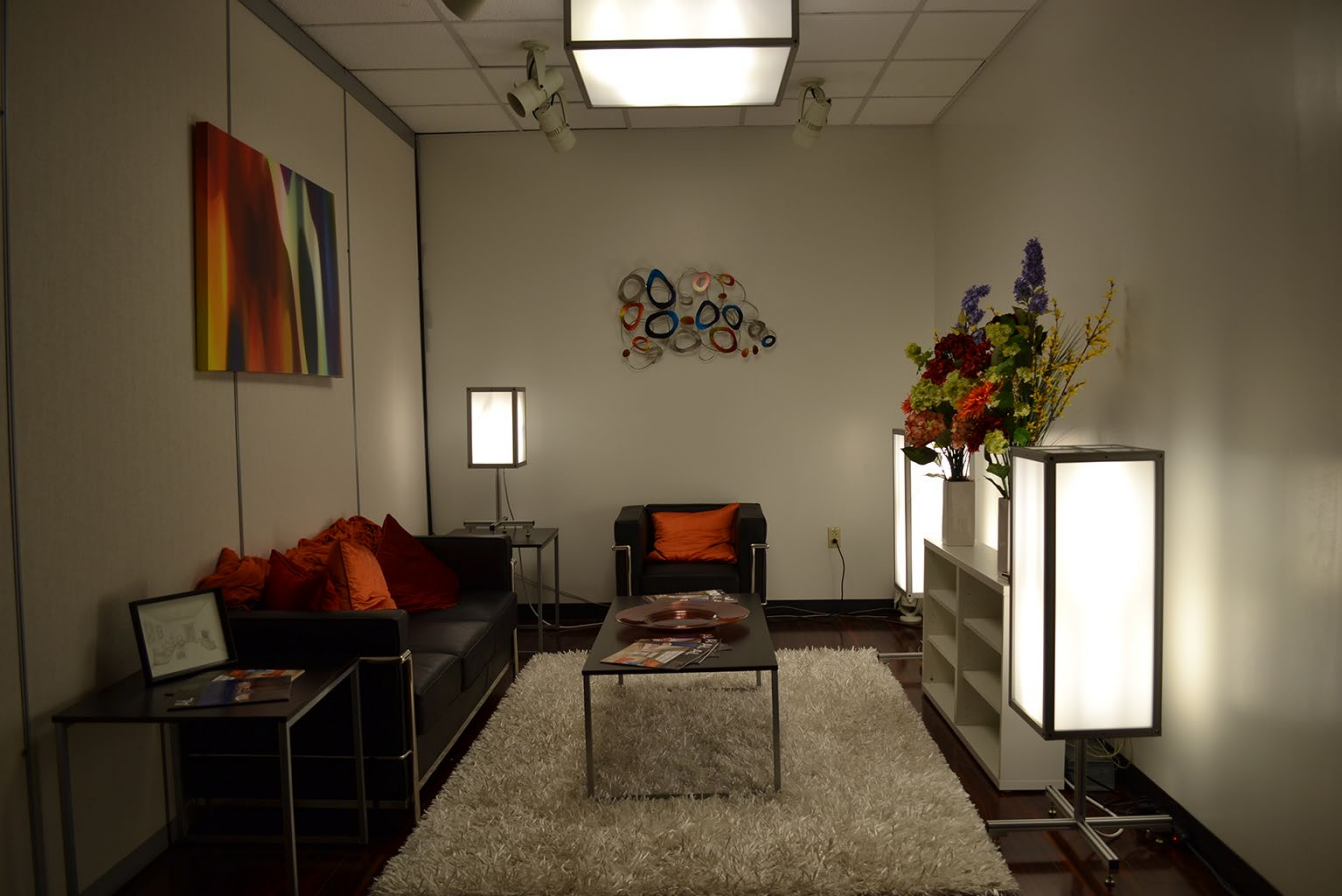
Full-scale residential prototype. The prototype included furnishings of a typical residential living room and was outfitted with a novel 5-channel color changing LED lighting system. The five channels included “red”, “green”, “blue”, “cool white”, and “warm white”. In this image, all 5 channels are on at 100% (ceiling mounted RGB spotlights are not on).

A full-scale, 150 square foot residential mockup was constructed in the lighting laboratory at Penn State University, University Park, Pennsylvania, USA and furnished as a modern residential living room containing a table, couch, chair, coffee table, end tables, and a bookcase/TV stand (Fig. 2). The space had three permanent, neutral gray walls; the room’s threshold had a moveable, felt blackout curtain that was closed during all measurements.

**Figure 3:**
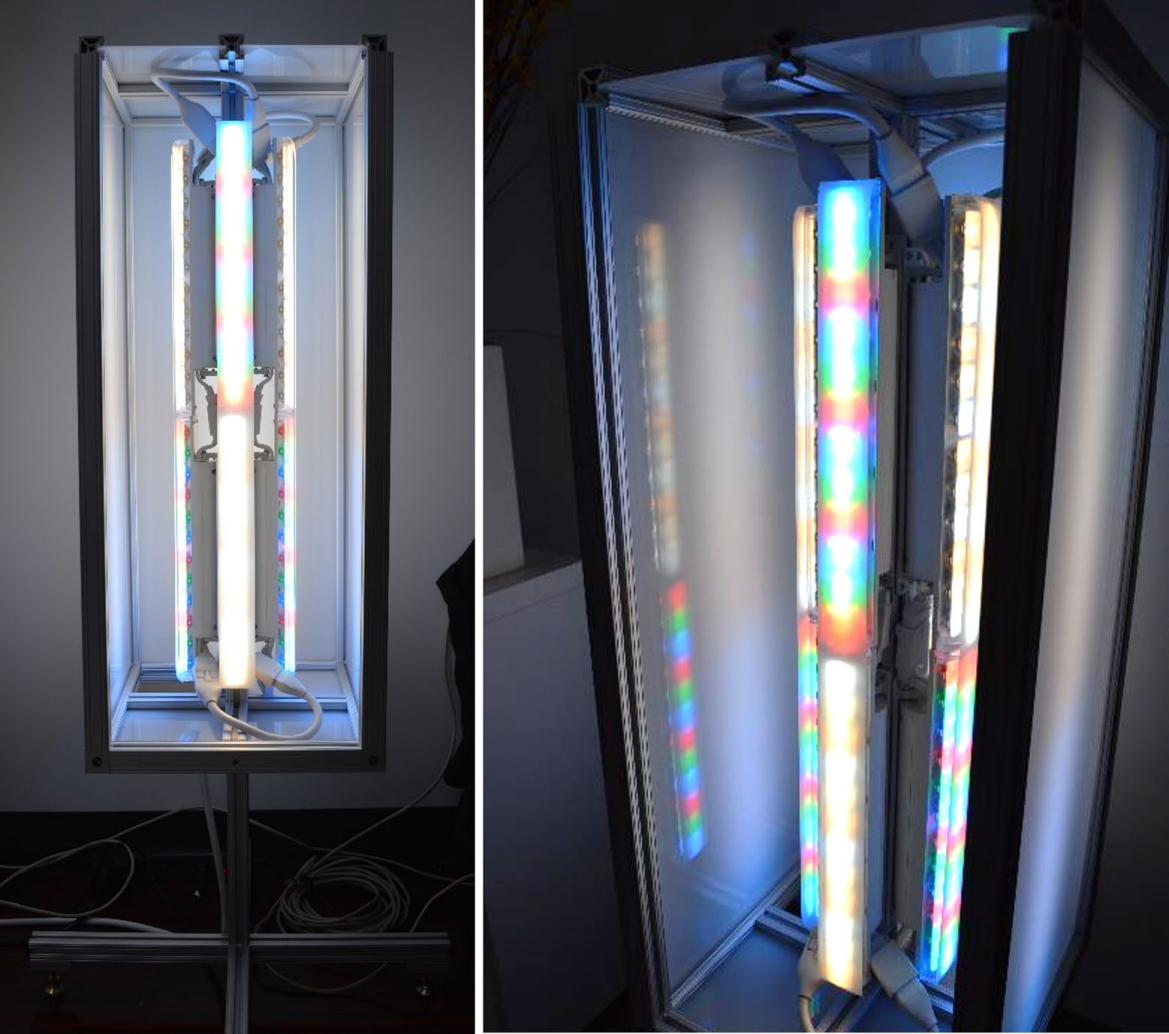
Typical luminaire assembly consisted of a series of Philips Powercore fixtures arranged around a central post and enclosed in a housing constructed of an aluminum frame with frosted acrylic panel inserts. Acrylic panels were used to blend the individual LEDs. For further diffusion, three individual strips of vellum were applied to the surface of each fixture.

The space was equipped with a color-changing architectural lighting system consisting of 5 individually controllable channels: nominally Red (“R”), Green (“G”), Blue (“B”), Warm White (“WW”), and Cool White (“CW”). Commercially available LED color-changing fixtures were organized into assemblies and enclosed in custom aluminum-framed housings with frosted acrylic diffusion panels. Within each assembly, three slits of vellum were applied to the surface of the individual LED strips for further diffusion and blending of the individual LED diodes (Fig. 3). Architectural spotlights were used to accent artwork. Diffusing sheets of velum and glare shields were used to maintain standards of visual quality for a residential interior. The lighting system was controlled via a Pharos Touch Panel Controller with a custom interface.

This work intentionally uses a real lighting system to demonstrate the variability that is easily achievable with lighting products available today, instead of probing the extremes of what may be achievable with an idealized spectral model. In any multi-channel LED system, the total number and chromaticity of the system’s channels, the power of each channel relative to one-another, and the peak wavelengths of the channels will impact the range of the achievable results. Nevertheless, the results presented in this manuscript represent a plausible range in CS and mel-EDI for light sources that are commercially available today.

### Measurement

Spectral measurements were taken with a calibrated PR-655 SpectraScan Spectroradiometer (Photo Research Inc., Cary, NC, USA) aimed at a diffuse reflectance standard (SRT-MS-100, *ρ* = 99%) (Labsphere North America, North Sutton, NH, USA). The spectroradiometer and the reflectance standard were positioned in-line, approximately 4 feet apart, with the reflectance standard located at the center of the room’s sofa and 3.5 feet above the floor. Illuminance measurements were taken with a Minolta T-10 illuminance meter (KONICA MINOLTA, Ramsey, NJ, USA) at the center of the room’s sofa, 3.5 feet above the floor. These measurements characterize light entering the eye of an occupant seated on the couch, looking forward. Measurements included direct and interreflected light and therefore incorporated the spectral reflectance and absorption of room surfaces.

**Figure 4:**
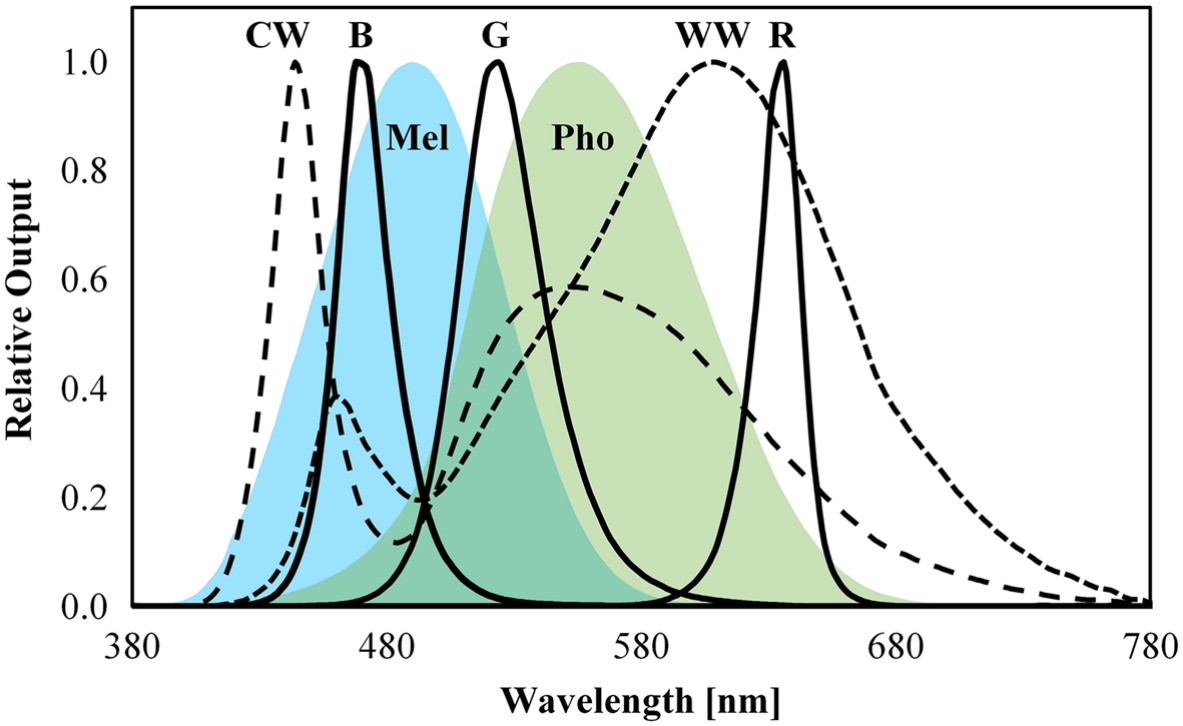
Relative spectral power distributions for the 5 channels of the lighting system. R, “Red”; G, “Green”; B, “Blue”; WW, “Warm White”; CW, “Cool White”; Pho,  photopic Luminous Efficacy Function and Mel , melanopsin action spectrum.

Illuminance and spectral measurements were taken at the plane of the eye for each of the 5 channels at 12 dimming levels (100, 90, 80, 70, 60, 50, 40, 30, 20, 15, 10, and 5% of full output) to permit reconstruction, via simulation, of the luminous conditions for all possible channel combinations. Illuminance measurements were used to determine a unique dimming curve for each channel. The relative spectral power distributions (SPDs) for the five channels are shown in Fig. 4.

### Simulation

To evaluate the relationship between CCT, CS, and mel-EDI a simulation was run using MATLAB® to generate a data set of many spectral combinations. The simulation proceeded as follows:

1. Generate a composite spectral power distribution (SPD) of the 5 channels using a randomly generated linear combination. The multiplier for each channel was randomly generated and the resulting channel scaling was determined using each channel’s unique dimming curve.
2. If the resulting composite SPD met all the following criteria, it was retained:
  - Chromaticity within ± 0.01 *D*_uv_ of the blackbody locus
  - CCT within ± 25 K of 6 practical CCTs: 2500, 3000, 3500, 4000, 5000, and 6000 K
3. Compute CS (at various illuminance levels), mel-DER, mel-EDI (at various illuminance levels), and various measures from IES TM-30-20^29^. Illuminance scaling was used to decouple the results from the performance of our specific lighting system, permitting generalization to lighting systems producing more or less lumens.
4. Repeat Steps 1–3, 30 million times. These data form the “Full Dataset”.
5. Create a subset of the data by filtering *D*_uv_ to eliminate all spectral combinations outside of the ANSI *Basic* quadrangles^18^ to represent commercially viable spectra architectural lighting. These data form the “Filtered Dataset”.
6. Further filter the dataset for IES TM-30-20 *R*_f_ ≥ 70, *R*_f_ ≥ 80, and *R*_f_ ≥ 90, for considering the impact of color fidelity on achievable CS and mel-EDI values.

Analyses in this paper considered only one aspect of color rendition, color fidelity, because it is a commonly specified performance parameter and it served here as a useful performance floor while considering the relationship between CCT and CS or mel-EDI. Importantly, other aspects of color rendition—such as vividness and color preference^29^, metameric uncertainty^30–35^, and color discrimination^36,37^—are relevant to applied lighting. These aspects of color rendition may themselves exhibit tradeoffs with CS and mel-EDI and these relationships should be studied directly.

## Results

**Figure 5:**
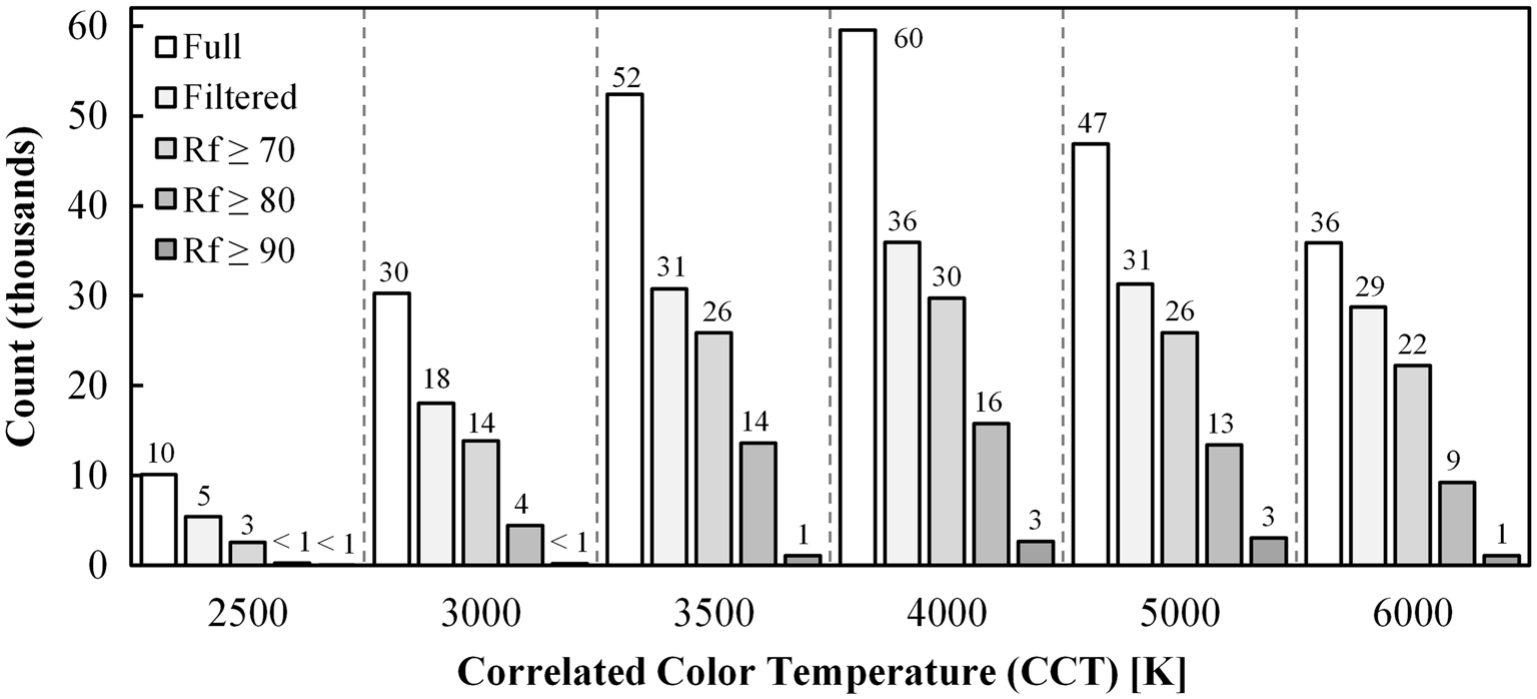
The number of composite SPDs retained at each of the nominal CCTs. The “Full” dataset includes SPDs with *D*_uv_ ± 0.01; the “Filtered” dataset includes SPDs with the *D*_uv_ tolerance of the ANSI *Basic* quadrangles. The other three series are color fidelity filters on the “Filtered” dataset. The nominal CCT with the most retained SPDs was 4000 K, with decreasing frequency at higher and lower CCTs.

A total of 235,039 composite SPDs met the criteria detailed in section “Simulation” before filtering *D*_uv_ to eliminate SPDs outside of the ANSI bins (simulation Steps 1–4); a total of 150,227 composite SPDs remained after filtering for inclusion in the ANSI bins (simulation Step 5). The most SPDs were retained at a CCT of 4000 K, and counts decrease with either increasing or decreasing CCT. Further filtering the “Filtered Dataset” for *R*_f_ ≥ 70, *R*_f_ ≥ 80, and *R*_f_ ≥ 90, as was done to evaluate the impact of color fidelity on achievable CS and mel-EDI, further decreased the number of retained SPDs. See Fig. 5.

### Melanopic DER and melanopic EDI

**Figure 6:**
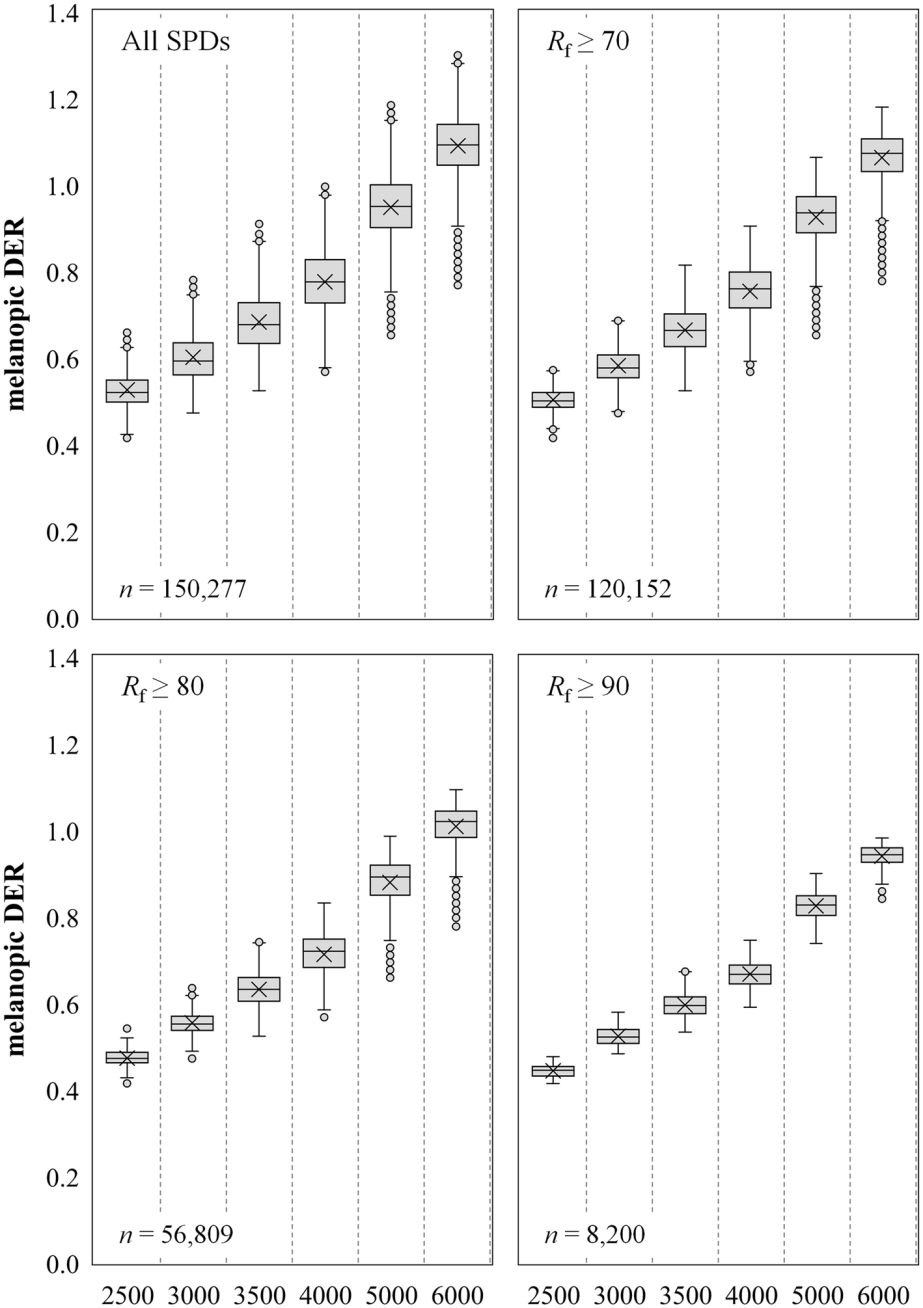
Boxplots of melanopic daylight efficacy ratio (mel-DER) as a function of nominal CCT. (Top left) All SPDs for the “Filtered” dataset. (Top right) The “Filtered” dataset further filtered for *R*_f_ ≥ 70. (Bottom left) The “Filtered” dataset further filtered for *R*_f_ ≥ 80. (Bottom right) The “Filtered” dataset further filtered for *R*_f_ ≥ 90. In all cases, the average mel-DER increases as CCT increases, but so too does the achievable mel-DER range. As the average fidelity floor is raised (i.e., from *R*_f_ ≥ 70 to *R*_f_ ≥ 80 to *R*_f_ ≥ 90), the average, maximum, and achievable range of mel-DER decreases.

Figure 6 shows boxplots for melanopic daylight efficacy ratio (mel-DER) for all retained SPDs by CCT for the Filtered Dataset. A clear trend between mel-DER and CCT is evident where, on average, mel-DER increases as CCT increases (*r*^2^ > 0.86). This trend is consistent with that demonstrated by Zandi et al.^38^, Carpentier and Meuret^39^, and Spitschan^40^. Summary statistics are provided in Table 1 and can be used to estimate mel-DER for an SPD based on CCT and IES *R*_f_.

**Table 1:**
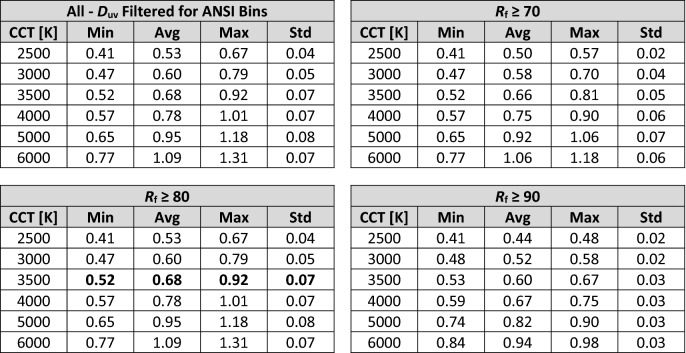
Statistical summary of melanopic daylight efficacy ratio^26^ for SPDs with various fidelity (*R*_f_) filtering. This table can be used as a quick reference to gauge the magnitude of mel-EDI for an SPD based on its nominal CCT and IES TM-30-20 *R*_f_. For example, a light source with a CCT of 3500 K and *R*_f_ near 80 will have an mel-DER between 0.52 and 0.75; See bolded values in table.

Notably, there is significant variation about the average mel-DER at any fixed CCT. For an SPD with a CCT of 3500 K and an *R*_f_ ≥ 80, a common lighting specification, mel-DER can vary between 0.52 and 0.75. At a photopic illuminance of 300 lx, a commonly specified illuminance level, this equates to mel-EDI values ranging from 157 to 226 lx. Alternatively, if a design target of mel-EDI = 218 lx is set—as might be done to achieve the maximum 3 points in WELL v2, “L03 Circadian Lighting Design”^27^—the spectrum alone can cause a required photopic illuminance between 289 and 416 lx. This is a difference of 127 photopic lux of required illuminance from the choice of spectrum alone.

Figure 6 also shows that as the color fidelity floor is raised (i.e., from *R*_f_ ≥ 70 to *R*_f_ ≥ 80 to *R*_f_ ≥ 90), the mean and spread of achievable mel-DER values decrease at any single CCT. This is consistent with the results of Carpentier and Meuret^39^. (Note that when color fidelity is maximal—i.e., IES TM-30 *R*_f_ = 100—the ranges shown in Fig. 6 reduce to a point because the SPD of the test source must be substantially identical to the SPD of the reference illuminant. In this case, mel-DER increases monotonically with CCT because the SPD at maximal color-fidelity varies smoothly and predictable with CCT.) Still, for an SPD with a CCT of 3500 K and an *R*_f_ ≥ 90, mel-DER can range from 0.53 to 0.67 (Table 1). At a photopic illuminance of 300 lx, for example, this equates to a range of mel-EDI values from 160 to 202 lx. Alternatively, if a target of mel-EDI = 218 lx is set—as might be done to achieve the maximum 3 points in WELL v2, “L03 Circadian Lighting Design”^27^—the spectrum alone can cause a required photopic illuminance between 323 and 409 lx. This is a difference of 88 photopic lux from the choice of spectrum alone, even when average color fidelity is very high.

Following (Eq. 2), Fig. 6 and Table 1 can be converted to mel-EDI values via multiplication by any photopic illuminance value. To convert Fig. 5, uniformly multiply the y-axis values by the designated illuminance. The same is true for values in Table 1. Figure 6 and Table 1 can be converted from mel-DER to Melanopic Ratio (MR) using (Eq. 4), then converted from MR to EML using (Eq. 5).

It is clear from (Eq. 2) and (Eq. 5) that both mel-EDI and EML scale linearly with photopic illuminance such that doubling photopic illuminance, for example, doubles mel-EDI and EML.

### Circadian stimulus

**Figure 7:**
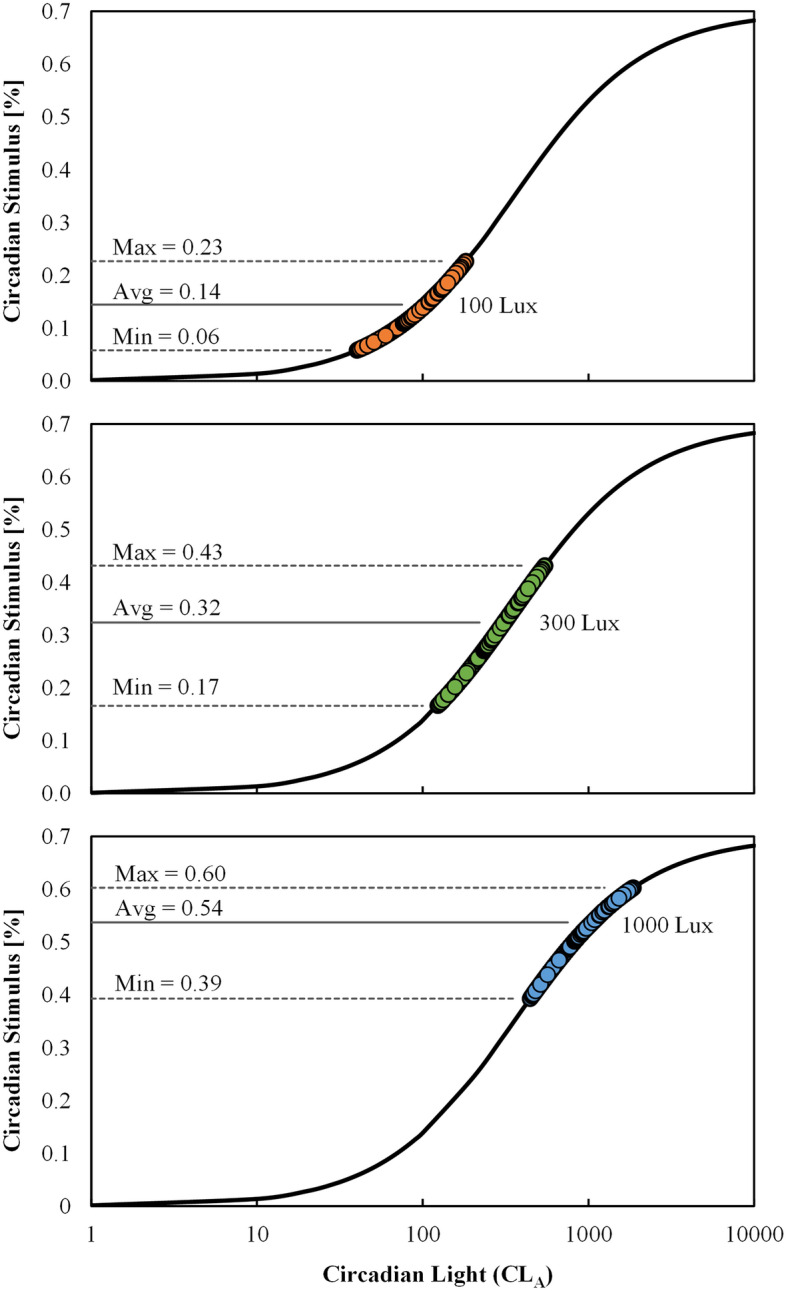
The range of Circadian Stimulus (CS) values as a function of Circadian light (CL_A_) for 100 lx (top), 300 lx (middle), and 1000 lx (bottom). At any fixed photopic illuminance, a substantial range of CS values can be achieved due to the choice of spectrum alone. The largest range occurs near 300 lx which falls near the inflect point of the CS sigmoid curve. Values are not sub-divided by CCT.

Figure 7 shows CS plotted against CL_A_ for photopic illuminances of 100 lx (top), 300 lx (middle), and 1000 lx (bottom). The achievable range of CS values decreases as illuminance departs from 300 lx, where the largest achievable CS range occurs. Figure 7 demonstrates the intensity-based dose–response relationship between CL_A_ and CS where intensity is first order information and spectrum is second order information. Said another way, intensity controls the order-of-magnitude of the effect, and spectrum creates variability about that point. Summary statistics for Fig. 7 are provided in Table 2 and can be used to estimate CS for an SPD based on corneal illuminance and IES *R*_f_.

**Table 2:**
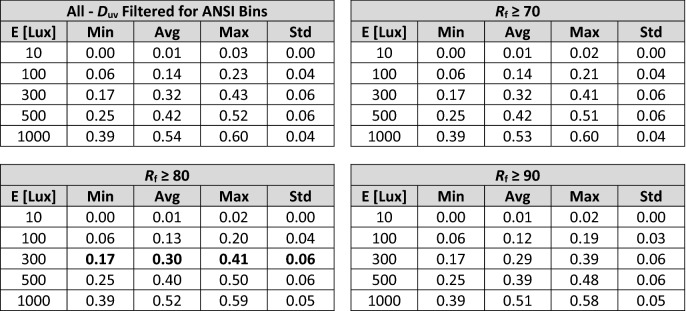
Statistical summary of Circadian Stimulus for SPDs with various fidelity (*R*_f_) filtering. This table can be used as a quick reference to gauge the magnitude of CS for an SPD based on its specified corneal illuminance (E_v_) and approximate *R*_f_. For example, a light source with an *R*_f_ of 81 at 300 lx is likely to have a CS between 0.17 and 0.41 (not considering CCT); See bolded values in table.

**Figure 8:**
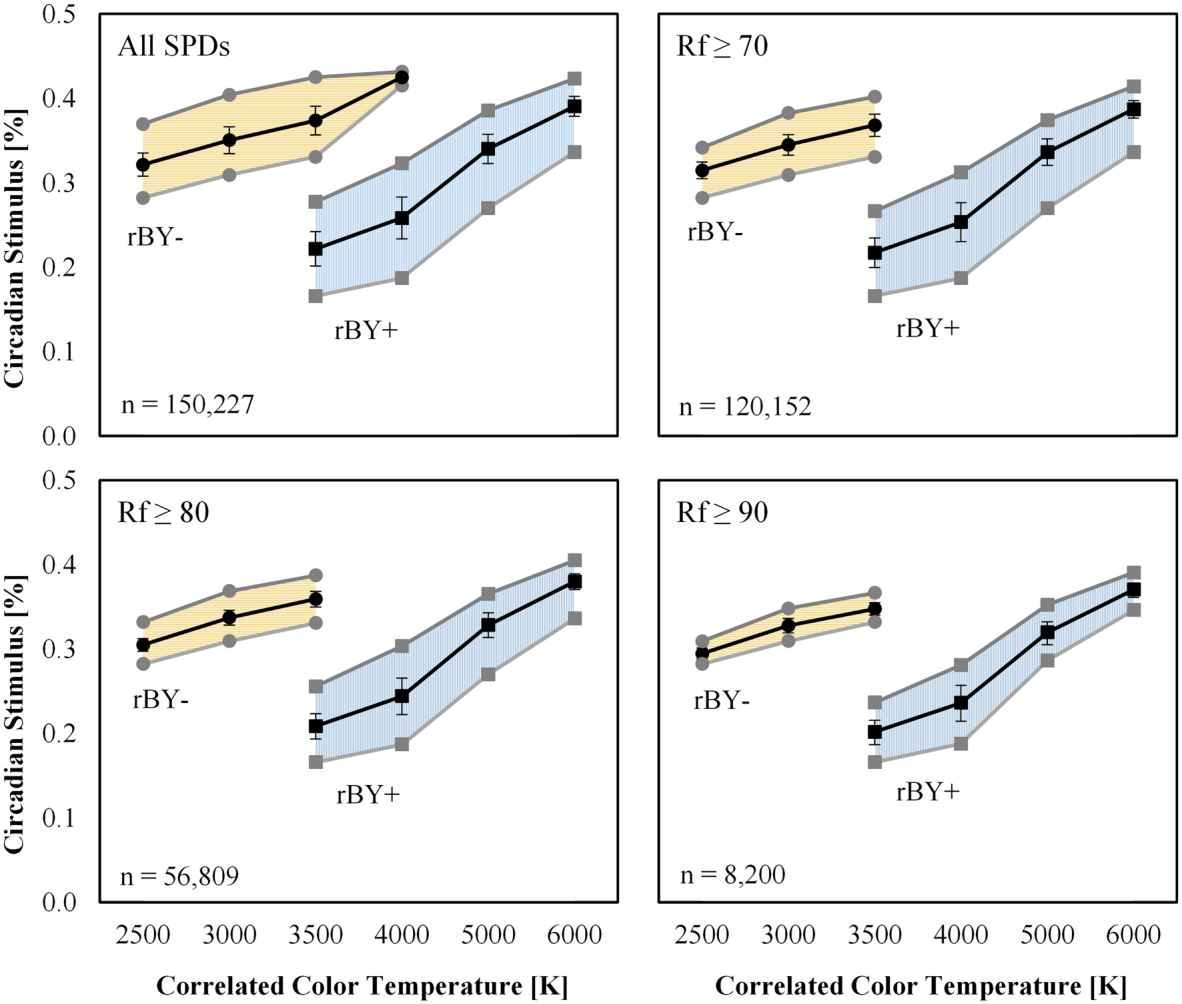
Circadian stimulus (CS) as a function of nominal CCT at a photopic illuminance of 300 lx. (Top left) All SPDs for the “Filtered” dataset. (Top right) The “Filtered” dataset further filtered for *R*_f_ ≥ 70. (Bottom left) The “Filtered” dataset further filtered for *R*_f_ ≥ 80. (Bottom right) The “Filtered” dataset further filtered for *R*_f_ ≥ 90. “rBY-” indicates the “yellow wins” scenario of the CS model (also indicated by the horizontal hatching and circle markers); “rBY+ ” indicates the “blue wins” scenario of the CS model (also indicated by the vertical hatching and square markers). Lines show the maximum (grey), minimum (grey), and average (black) CS within each nominal CCT; error bars indicate the standard deviation. On average, sources with rBY- have higher CS values than sources with rBY+. The b-y spectral opponency causes the observable discontinuity near 3500 K where the model switches from one hypothetical model of phototransduction to another. As the average fidelity floor is raised (i.e., from *R*_f_ ≥ 70 to *R*_f_ ≥ 80 to *R*_f_ ≥ 90), the achievable CS range decreases.

**Figure 9:**
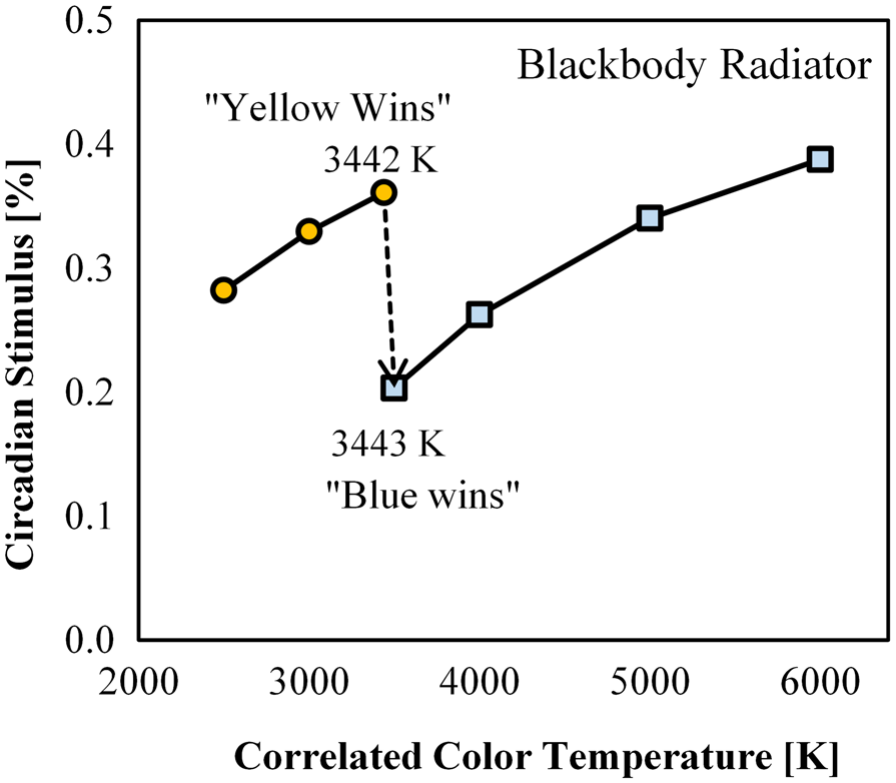
Circadian Stimulus (CS) as a function of CCT for a blackbody radiator. The exact inflection point of the b-y spectral opponency of the CS model for a blackbody radiator occurs between 3442 and 3443 K. Up to 3442 K, “yellow wins” (indicated by circle markers); from 3443 K onwards, “blue wins” (indicated by square markers). The dashed line indicates the transition point of the model and is dashed to indicate that this transition is not continuous (values along this line are not achievable) and that the CS model is a step function.

**Figure 10:**
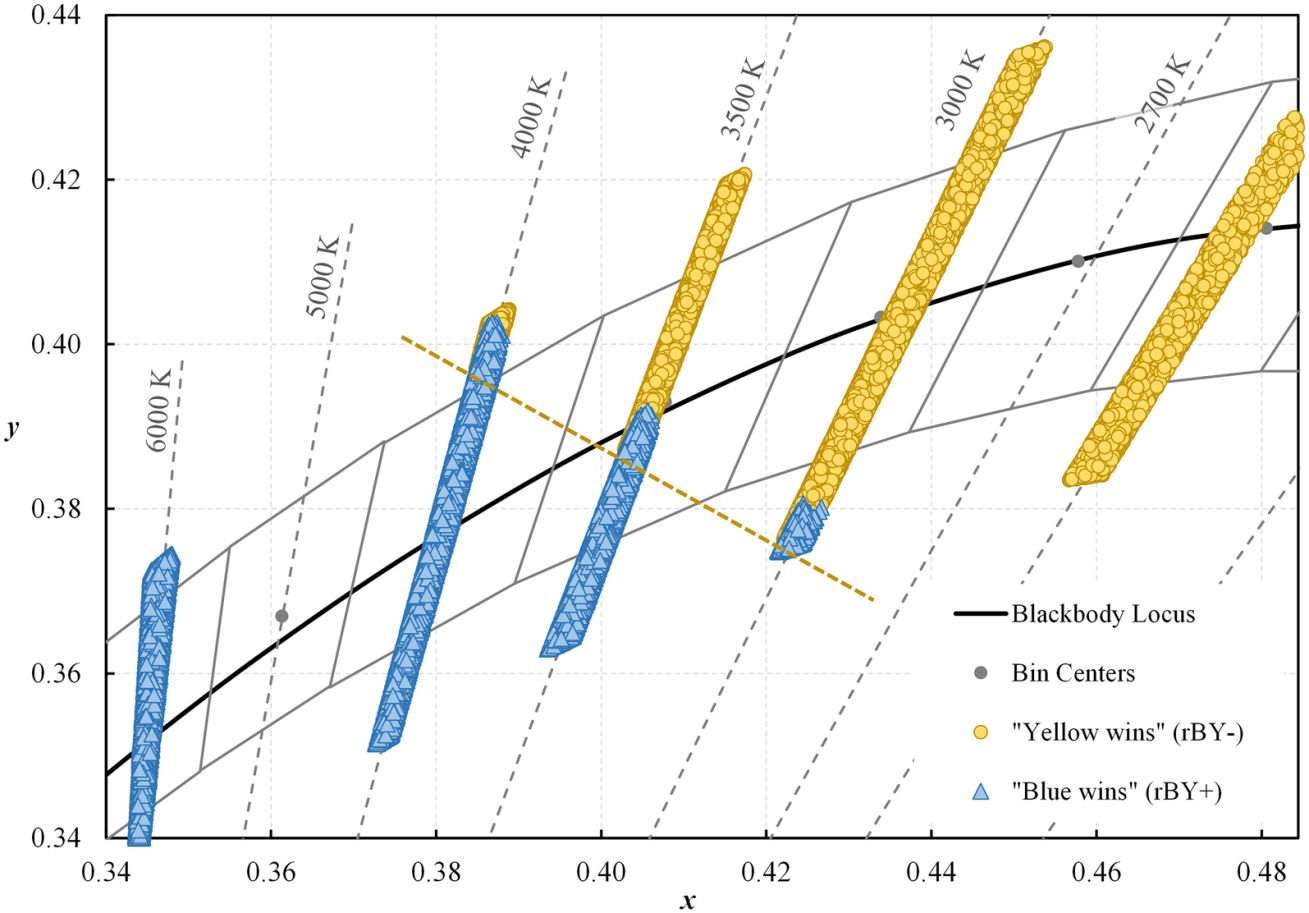
Chromaticity of the “Full” dataset, filtered for *R*_f_ ≥ 70, plotted in the CIE 1931 (*x*,*y*) chromaticity diagram. The b-y spectral opponency of the CS model manifests strongly in chromaticity space cutting the 3500 K and 4000 K *Basic* ANSI quadrangles diagonally. The yellow dashed line indicates the limit of the “yellow wins” dataset (circle markers) which is hidden by the “blue wins” dataset (triangle markers). When this line representing the boundary of the b-y spectral opponency is extended, it intersects the spectrum locus near 500 nm which is near the crossover wavelength of the functions that the CS model uses to determine “blue wins” (rBY+) or “yellow wins” (rBY-).

At a fixed illuminance of 300 lx, Fig. 8 shows that the range of achievable CS varies significantly as a function of CCT. Summary statistics are provided in Table 3 and can be used to estimate CS based on CCT and spectral-opponency. At 2500 K and 3000 K, “yellow wins” (rBY-) for all SPDs due to the prevalence of long wavelength radiation in the composite SPDs. Near 3500 K a battle between “blue wins” (rBY+) and “yellow wins” (rBY-) is apparent leading to an observable discontinuity. (Note that the exact inflection point of the CS model is between 3442 and 3443 K for a blackbody radiator, see Fig. 9, and is strongly dependent on chromaticity, see Fig. 10). By 4000 K, “blue wins” (rBY+) for all SPDs, though CS is noticeably reduced compared to the “yellow wins” scenarios.

**Table 3:**
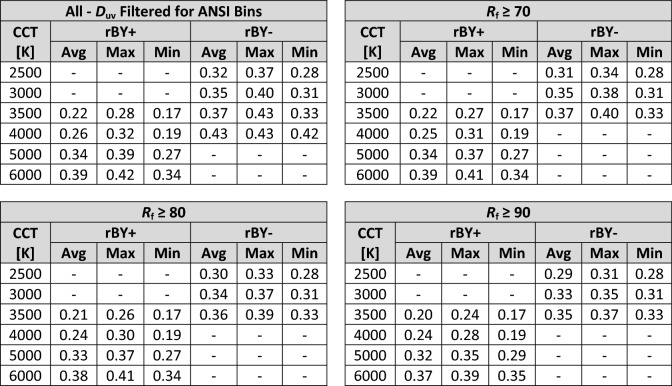
A summary of circadian stimulus as a function of CCT with various *R*_f_ filtering. rBY+ indicates "blue wins". rBY- indicates "yellow wins" and is determined by the balance of spectral content relative to 500 nm, approximately.

The spectral opponency of the CS model—observed most clearly at the discontinuity near 3500 K—has a large impact on the resulting CS value. For SPDs with *R*_f_ ≥ 70, the lowest CS at 2500 K (0.28) has a higher CS than the highest CS at 3500 K and rBY+ (0.27). This holds true and is exacerbated as the dataset is filtered for increasing color fidelity—e.g., *R*_f_ ≥ 80 and *R*_f_ ≥ 90—and the achievable CS range at a fixed CCT decreases.

**Figure 11:**
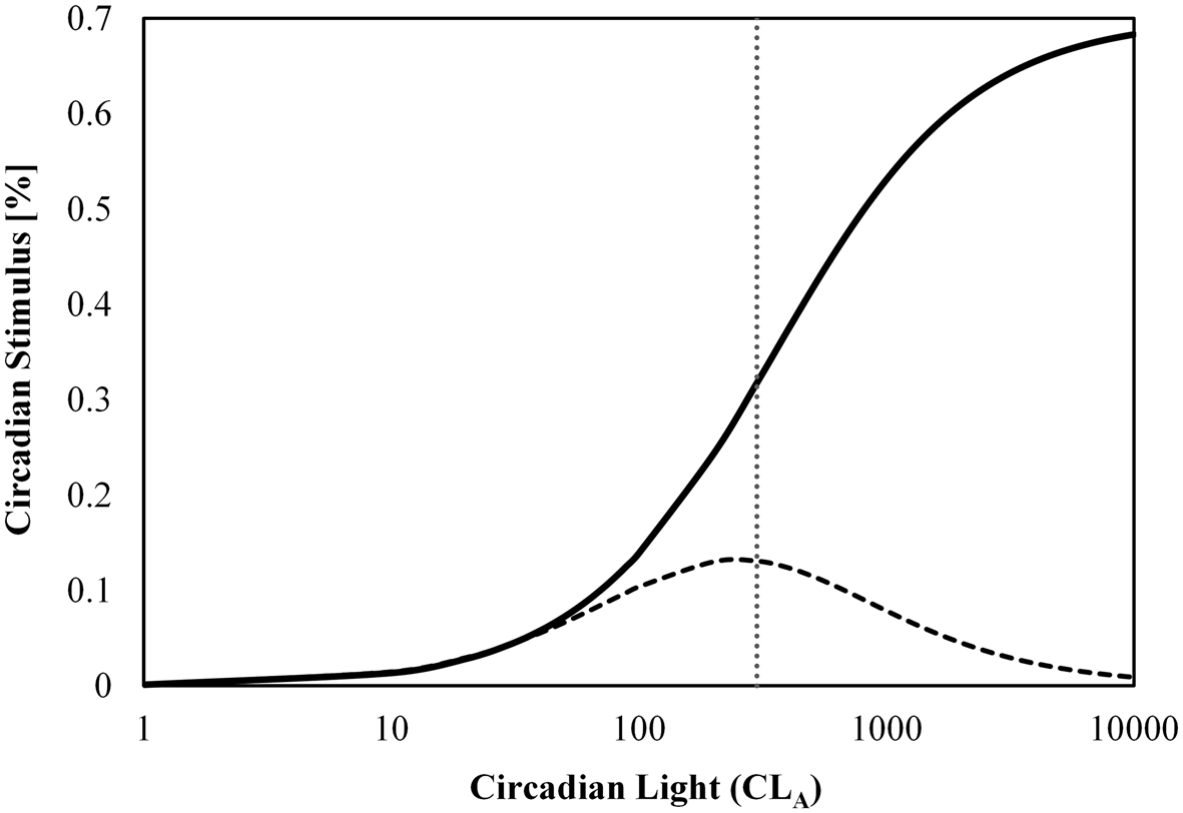
The thick black line shows the four-parameter logistic function that determines Circadian Stimulus (CS) from Circadian Light (CL_A_). The dashed curve indicates the difference in CS when CL_A_ is doubled. This can be thought of the “strength” of doubling CL_A_ on CS at the specified CL_A_ value. The vertical dashed line indicates that the largest increase on CS of doubling CL_A_ occurs at a CL_A_ of 300 (near the inflection point of the logistic function), which can be for a photopic illuminance of 300–500 lx, depending on spectrum.

CL_A_ scales linearly with photopic illuminance such that doubling photopic illuminance, for example, doubles CL_A_. CL_A_ then maps non-linearly onto CS via the four-parameter logistic function observable in Fig. 8. Figure 11 shows the impact of doubling CL_A_ (i.e., doubling photopic illuminance) on the resulting CS value. The largest benefit of doubling photopic illuminance occurs when the starting CL_A_ is near 300 (for photopic lux between 300 and 500 lx, depending on the spectrum) because it is near the inflection (or “threshold”) of the CS logistic function.

In general, for a given opponent pathway (rBY+ or rBY-), the maximum achievable CS at a fixed CCT increases as CCT increases, but composite spectra at a given CCT exhibit a wide range of CS values. At a fixed illuminance and CCT, CS can range by more than 0.1 (which is one-third (33%) of the daytime CS minimum of 0.3 recommendation of UL 24480^24^) and more than 0.2 at a CCT of 3500 K depending on the b-y spectral opponency (which is two-thirds (66%) of the daytime CS minimum of 0.3 recommendation of UL 24480^24^).

### Comparing CS and mel-EDI

To simplify lighting practice, lighting practitioners would understandably like a simple conversion between measures like CS and measures like mel-EDI (see, for example, Stodola^41^). We should first mention, however, that an apples-to-apples comparison between CS and mel-EDI is not possible. CS and mel-EDI are similar in that they both deal with spectral sensitivity, but they are different in that mel-EDI is based on a single photopigment and CS endeavors to account for the physiology of the eye/brain system. In support of this substantial theoretical difference, we quantitatively demonstrate the manner and degree of differences between mel-EDI and CS, thereby objectively substantiating their lack of comparability.

**Figure 12:**
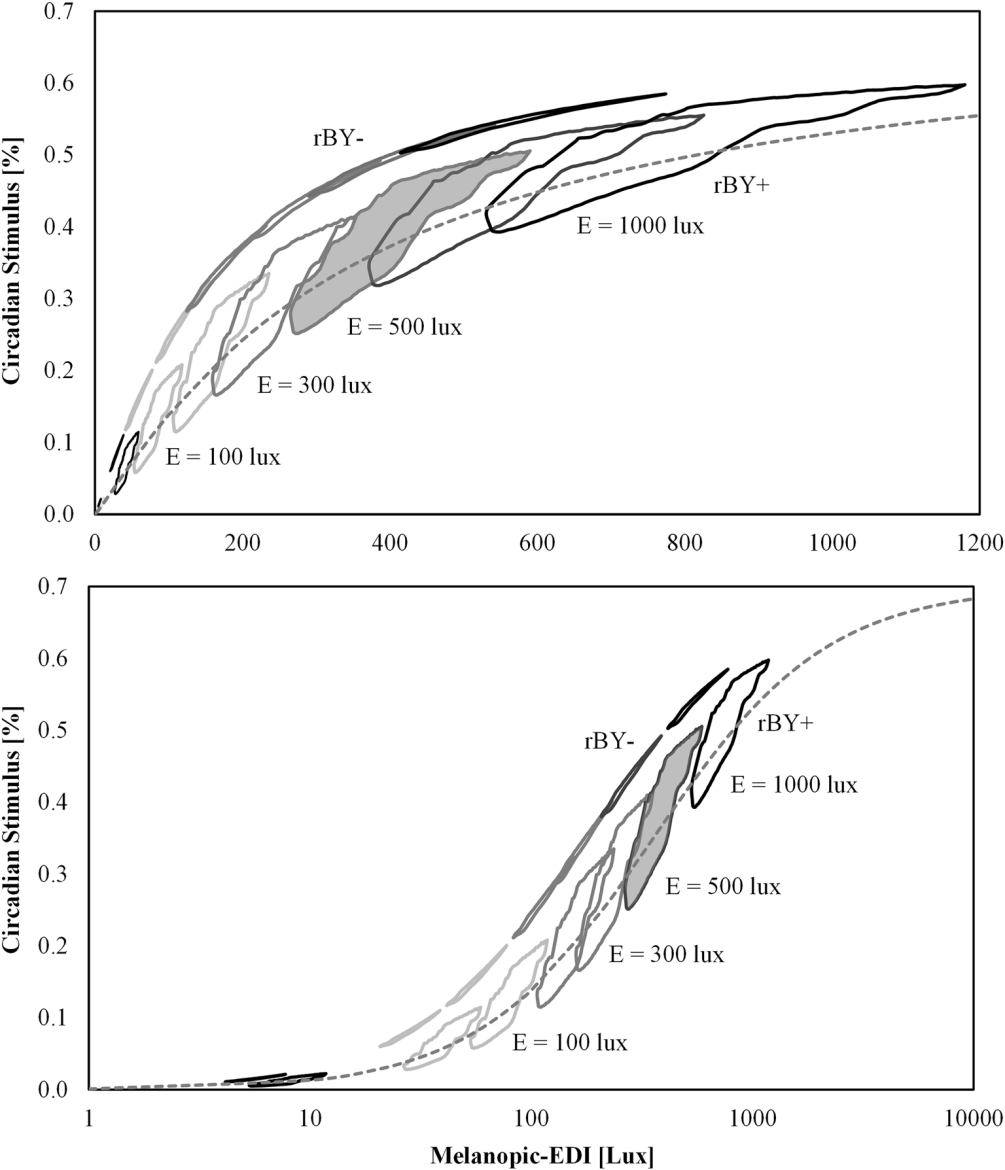
Circadian Stimulus (CS) as a function of melanopic equivalent daylight illuminance (mel-EDI) with sub-divisions for photopic illuminance represented as boundaries of the data. (Top) Linear scaling for the x-axis. (Bottom) Logarithmic scaling for the x-axis. Overall, there is a wide range of achievable CS values at a fixed mel-EDI and a strong discontinuity due to the b-y spectral opponency of the CS model (“yellow wins” indicated by rBY-, and “blue wins” indicated by rBY+). No simple or satisfactory mathematical conversion between CS and mel-EDI can be offered. The grey shaded area indicates the sub-division of the data with an illuminance of 500 lx, which is enlarged in Fig. 13.

Figure 12 compares CS and mel-EDI, with sub-divisions for illuminance, and demonstrates that no such easy mathematical conversion between CS and mel-EDI can be offered when a spectrally diverse set of SPDs is considered. For example, at a photopic illuminance of 300 lx and a mel-EDI of 218 lx (a value achieving the maximum 3 points in WELL v2), CS varies between 0.22 and 0.39 (0.22 and 0.32 for rBY+ between CCTs of 3500 and 5000 K, and 0.38 and 0.39 for rBY- at a CCT of 3500 K).

**Figure 13:**
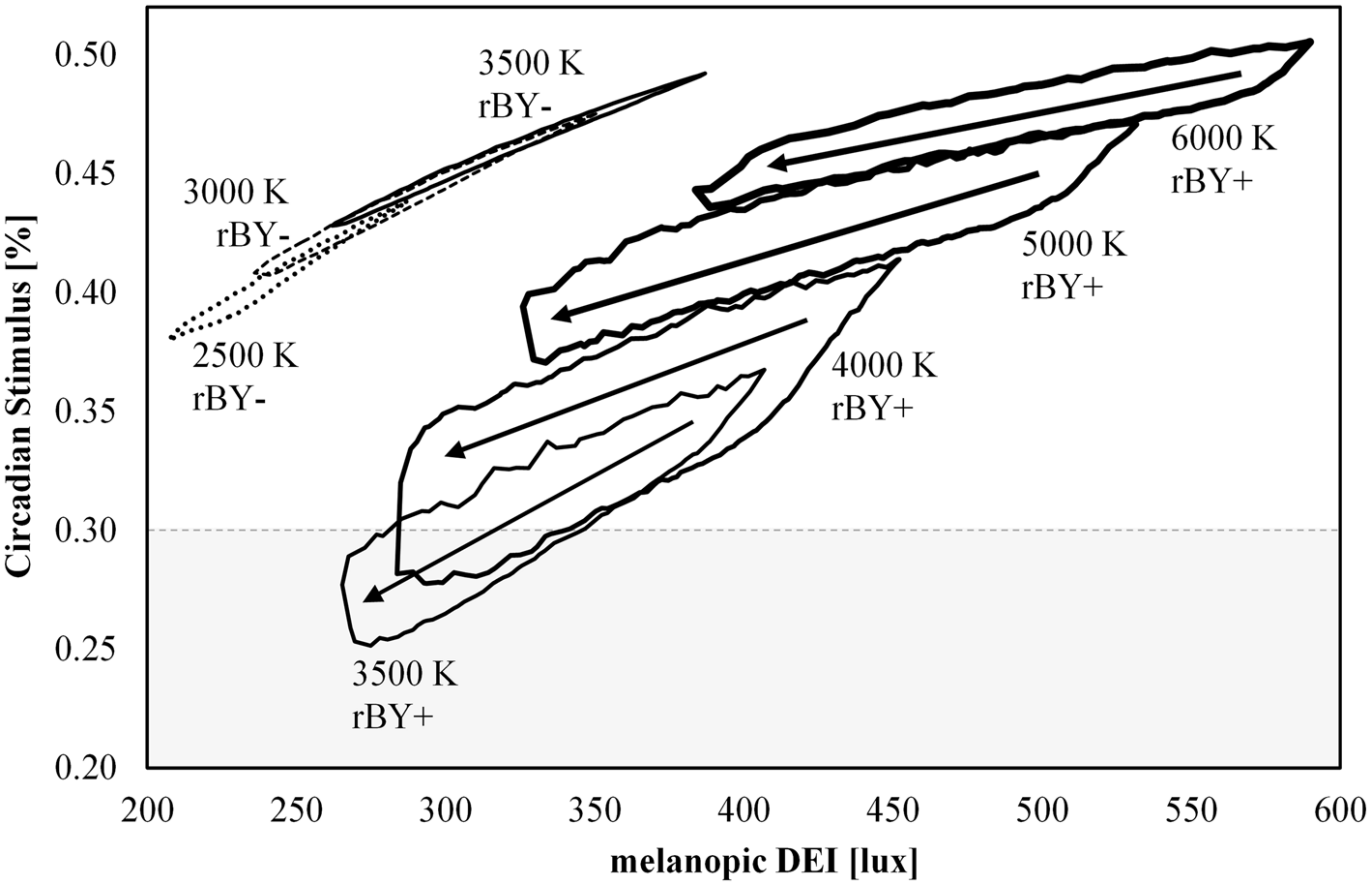
Circadian Stimulus (CS) as a function of melanopic equivalent daylight illuminance (mel-EDI) at a photopic illuminance of 500 lx enlarged from Fig. 12 with the addition of subdivisions for nominal CCT. Arrows show, on average, increasing *R*_f_. Zooming in on the relationship at a fixed illuminance illustrates the large range of achievable CS values at a fixed mel-EDI that is driven by spectral configuration and partly driven by the discontinuity between “blue wins” (rBY+) and “yellow wins” (rBY-) of the CS model. The horizontal grey dashed line indicates the daytime CS recommendation of CS ≥ 0.3 of UL 24480.

Figure 13 makes this point more forcefully, showing the relationship between CS and mel-EDI at a fixed illuminance of 500 lx with CCT sub-divisions. Visibly, no best-fit equation could satisfactorily describe this relationship. Even if tempted to build best-fit relationships for CCT subgroups, CS can vary between 0.26 and 0.45 for CCT = 3500 K, and from 0.28 to 0.35 for CCT = 4000 K, each at a fixed mel-EDI of 300 lx. Even if such a range is acceptable, or a simple relationship could be found, for example by inappropriate selective paring of the dataset or analyzing only broadband phosphor-converted LEDs, the conceptual and computational differences between measures like CS and measures like mel-EDI make such a conversion inappropriate.

Figure 13 is useful for evaluating a recommendation of UL 24480^24^ which states that without regard to spectrum, a photopic illuminance of 500 lx can be specified to achieve the daytime recommendation of CS ≥ 0.3. They indicate that at 500 lx, “90% of commercially available LEDs with CCTs between 2700 and 6500 K will meet the circadian-effective light design criterion of CS ≥ 0.3.” In this analysis, 97% of SPDs at a photopic illuminance of 500 lx fall above the CS ≥ 0.3 criteria. Most of the SPDs with CS < 0.3, however, are at 3500 K and high color fidelity. This is a common lighting specification and is therefore potentially problematic.

UL 24480 also makes recommendations of CS ≤ 0.2 and CS ≤ 0.1 in the evening and night, respectively. In this study, 99.5% of SPDs at 100 photopic lux had CS ≤ 0.2 (only 28.5% of SPDs had CS ≤ 0.2 at 200 photopic lux). Without regard to spectrum, a photopic illuminance of 100 lx can be reasonably assumed to comply with UL 24480’s evening recommendations. For the nighttime recommendation, 88.3% of SPDs at 50 photopic lux had CS ≤ 0.1 (only 22.8% of SPDs had CS ≤ 0.1 at 100 photopic lux). Without regard to spectrum, a photopic illuminance *less than* 50 lx can be reasonably assumed to comply with UL 24480’s nighttime recommendations.

## Discussion

### Predicting biological potency with CCT fails in concept

CCT describes the visual “warmness” or “coolness” of the color appearance of nominally white light. Melanopic-EDI is a measure of the stimulation of the ipRGC photoreceptors via the action spectra of their contained photopigment melanopsin. Circadian Stimulus is a model of human nocturnal melatonin suppression as a function of light source spectrum and intensity. That CCT is a measure of visual perception should immediately raise doubts about its ability to predict any measure that is not related to the visual perception of nominally white light. Furthermore, that two metameric spectra, thus having the same CCT, can have largely different quantities of melanopsin-stimulating radiation make it obvious that any simple relationship that can be found between CCT and CS or mel-EDI is merely a coincidence or the result of selective paring of the dataset.

There is currently incomplete knowledge about how photoreceptor signals are combined and processed within the retina (e.g., by ipRGCs that combine the intrinsic melanopic response with extrinsic signals from cones and rods) and incomplete understandings of how those signals are then processed by the brain. As such, CIE S026 does not propose a working model of the eye-brain mechanism. This is because quantifying individual photoreceptor responses is a means to an end, not the end itself. CIE S026 acknowledges this: “If…the relative photoreceptor inputs to any response under defined conditions could be resolved it would be possible to predict the magnitude of evoked [non-visual] responses from the combination of the effective light intensity for each of the individual photoreceptors.” CIE S026 represents a significant achievement by standardizing quantification of photopigment action spectra that serve as inputs into the eye-brain mechanism. In this conceptual framework, CIE S026 provides the foundation upon which to build mechanistic models. We are reluctant about models that are over reliant on any single photopigment. Whether mel-DER becomes an input measure into mechanistic models or is proposed as a direct predictor of outcome measures (e.g., see Brown^42^), one thing is for certain: there is no expectation that models used to predict human non-visual response to light will make use of CCT.

### Predicting biological potency with CCT fails in practice

No numerical justification for the use of CCT as a predictor of biological potential can be offered. Figures 6, 8, 12, and 13 demonstrate that a sufficiently large variation in CS and mel-EDI exists at any fixed CCT and photopic illuminance. At 300 photopic lux, 3500 K, and a minimum *R*_f_ of 70—a common lighting specification—CS can range, albeit discontinuously, from 0.17 to 0.4 (a difference of 0.23), due to spectrum alone. This difference is more than two-thirds (66%) of the minimum daytime CS of 0.3 recommended by UL 24480^24^. At a design target of mel-EDI = 200 lx, 3500 K, and a minimum *R*_f_ of 70, spectrum can cause a required illuminance between 370 and 576 lx, a difference of 206 photopic lux due to spectrum alone.

Given the relative ease with which mel-DER and mel-EDI can be computed, no practical justification can be offered either. CCT is derived from the same information needed to compute mel-DER: the light source’s spectral power distribution. If the SPD is readily available for the calculation of CCT, mel-DER can be computed with a few additional computations. (Note that relative to calculations of mel-DER using a light source’s SPD, field measurements of mel-DER will likely differ due to attenuation of short wavelengths by room surfaces^43–49^; preliminary evidence suggests that field measurements of mel-DER are likely to be lower than estimates with a light source’s SPD^48–50^, especially where non-white, non-blue surfaces are present^51^).

Mel-EDI requires an estimate or measurement of illuminance. Estimating illuminance is common in lighting practice. In the field, measuring illuminance for the mel-EDI computation does not require an additional measurement device. Field measurements of mel-EDI provide the most accurate estimates and are recommended whenever possible.

Determining mel-DER and/or mel-EDI is relatively straightforward using testing and measurement procedures already common in lighting practice. In practice, shorthand rules can be useful to simplify illuminating engineering but predicting biological potential from CCT fails in theory and in execution. Simply, CCT should not be used as a proxy for the biological potency of light.

### CS model implications for spectral design

The opponent pathway in the CS model has a significant effect on resulting CS values whereby it is advantageous to the goal of maximizing CS, at a fixed illuminance, to do one of three things:

1. Force “yellow wins” (rBY-) by targeting nominal CCTs below 3500 K—or an actual CCT less than 3443 K for broadband spectra near the blackbody locus and with high color fidelity, or
2. target a positive *D*_uv_ when CCT is near 3500 K (increasing the likelihood of “yellow wins”, rBY-), or
3. target nominal CCTs greater than 4000 K (not including 4000 K).

To minimize CS, it is advantageous, at a fixed illuminance, to target a nominal CCT of 4000 K, or a nominal CCT of 3500 K if “blue wins” (rBY+) can be forced. Figure 10 shows that at a CCT of 3500 K, targeting chromaticities below the blackbody locus increases the likelihood of forcing rBY+. At a fixed illuminance, the CS model suggests that when the goal is to suppress the least amount of melatonin—for example, in the evening before sleep—light sources with a nominal CCT of 4000 K or 3500 K (with rBY+) should be targeted. These results contradict prevailing wisdom that light with proportionally more short wavelength radiation (that is, near the sensitivity of the melanopsin action spectra) has greater biological potency.

**Figure 14:**
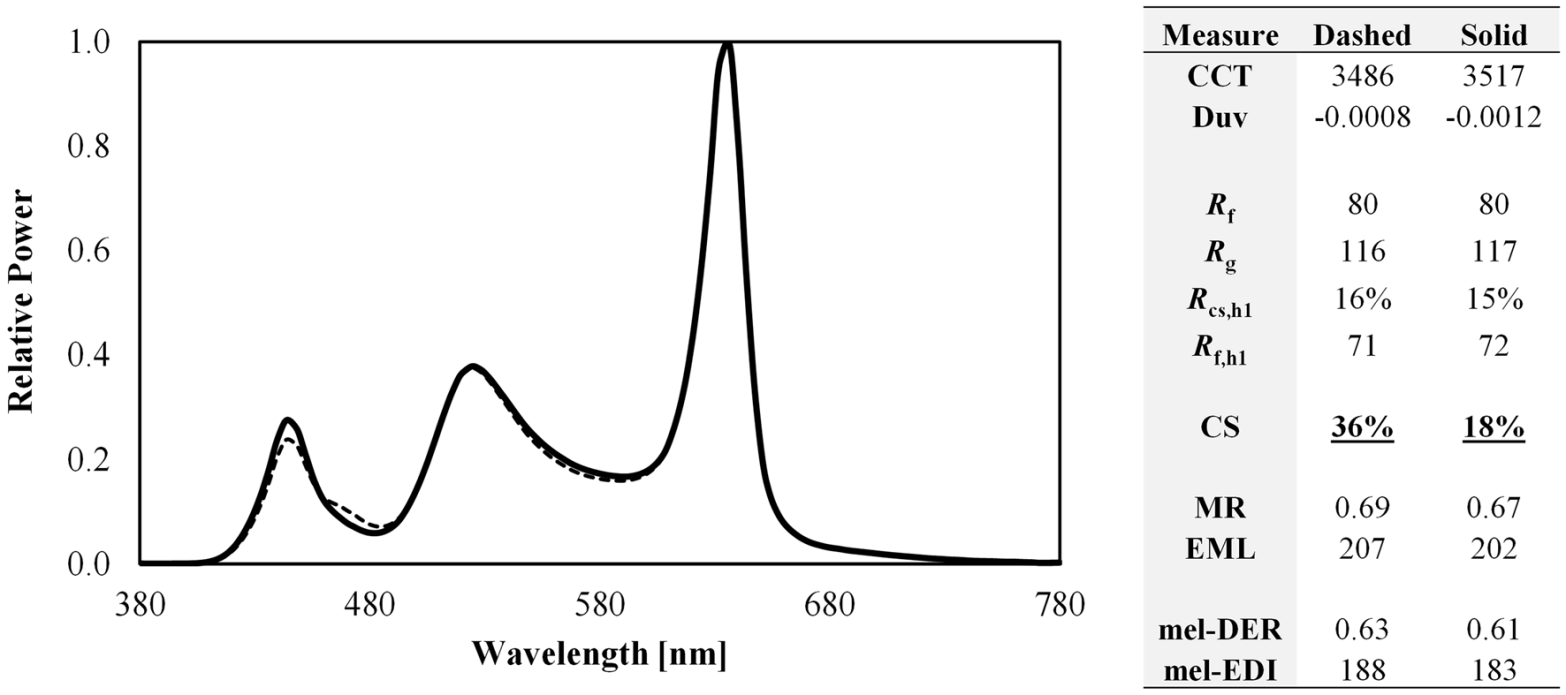
Two spectral power distributions with nearly identical spectral curves (left) but with a CS differing by 100% at a photopic illuminance of 300 lx (right). Because the SPDs are nearly overlapping, the dashed SPD may be difficult to see. For orientation, the largest spectral differences occur near 450 and 480 nm.

As shown in Fig. 10, the b-y spectral opponency of the CS model cuts diagonally across the 3500 K ANSI bin. This raises an important practical consideration. For manufacturers creating products near this boundary—as many do since products at 3500 K near the blackbody locus are popular—normal production tolerances could mean that some products within a single production run are rBY+ and some are rBY-, leading to a difference in CS of up to 0.23 at a photopic illuminance of 300 lx and *R*_f_ ≥ 70. This is a variation equal to two-thirds (67%) of the UL 24480 daytime recommendation of CS ≥ 0.3^24^ for light sources that have negligibly different SPDs (see Fig. 14). To avoid this conflict, manufacturers would need to push their products above the blackboy locus to maximize CS (forcing “yellow wins”), which may lead to less-preferred (and green-tinted) illumination^52–54^, or below the blackbody locus to minimize CS (to force “blue wins”).

From an engineering perspective, this discontinuity is peculiar and raises suspicion about the face validity of the CS model. Is the sharp discontinuity physiologically plausible, or simply an artifact of the model? If such opponency is physiologically present in normal observers, what is the role of observer variability (e.g., to what degree could an illuminant that is rBY+ to one observer be rBY- to a second observer, a question that is relevant in consideration of prior work about interindividual variability to light^55^)? Regardless, this discontinuity is pertinent to the spectral design of light sources, if CS values are important, with static or dynamic spectra near the chromaticity inflection demonstrated in Fig. 10, since there can be large variability in CS for products at 3500 K which have nearly identical performance on all other spectrally derived quantities.

## Conclusions

The lighting industry is experiencing rapid transformation as we expand our awareness of the non-visual impacts of light on humans. It is pertinent that we develop measures, methods, and strategies for implementing architectural lighting solutions that support these non-visual impacts. To do so, we need accurate and predictive measures of the biological potency of light that are based on sound science. In this study, we have argued that CCT is conceptually inappropriate for this purpose and performed a numerical analysis demonstrating that significant variation in circadian stimulus and melanopic equivalent daylight illuminance exists at any fixed CCT and photopic illuminance, making CCT an inappropriate proxy of those measures. Using CCT as a proxy for the biological potency of light cannot be justified.

## Data Availability

Data underlying the results presented in this paper are not publicly available at this time but may be obtained from the authors upon reasonable request. Inquiries should be directed to TE.
